# Identifying cost-based quality and performance indicators for home care: a modified delphi method study

**DOI:** 10.1186/s12913-024-11299-z

**Published:** 2024-07-24

**Authors:** Max Jajszczok, Cathy A. Eastwood, Mingshan Lu, Ceara Cunningham, Danielle A. Southern, Hude Quan

**Affiliations:** 1 Community Health Sciences Cumming School of Medicine, University of Calgary, 3280 Hospital Drive NW, Calgary, AB T2N 4Z6 Canada; 2 Departments of Economics and Community Health Sciences, University of Calgary, 2500 University Dr NW, Calgary, Alberta T2N1N4 Canada; 3 Centre for Health Informatics, University of Calgary, 3280 Hospital Drive NW, Calgary, AB T2N 4Z6 Canada

**Keywords:** Delphi method, Home health care, Quadruple AIM, Financial measurement, Quality indicators

## Abstract

**Background:**

This study, part of a multi-study program, aimed to identify a core set of cost-based quality and performance indicators using a modified Delphi research approach. Conceptually, this core set of cost-based indicators is intended for use within a broader health system performance framework for evaluating home care programming in Canada.

**Methods:**

This study used findings from a recently published scoping review identifying 34 cost-focused home care program PQIs. A purposive and snowball technique was employed to recruit a national panel of system-level operational and content experts in home care. We collected data through progressive surveys and engagement sessions. In the first round of surveying, the panel scored each indicator on Importance, Actionable, and Interpretable criteria. The panel set the second round of ranking the remaining indicators’ consensus criteria. The panel ranked by importance their top five indicators from operational and system perspectives. Indicators selected by over 50% of the panel were accepted as consensus.

**Results:**

We identified 13 panellists. 12 completed the first round which identified that 30 met the predetermined inclusion criteria. Eight completed the ranking exercise, with one of the eight completing one of two components. The second round resulted in three PQIs meeting the consensus criteria: one operational and two systems-policy-focused. The PQIs: “Average cost per day per home care client,” “Home care service cost (mean) per home care client 1y, 3y and 7y per health authority and provincially and nationally”, and “Home care funding as a percent of overall health care expenditures.”

**Conclusions:**

The findings from this study offer a crucial foundation for assessing operational and health system outcomes. Notably, this research pioneers identifying key cost-based PQIs through a national expert panel and modified Delphi methodology. This study contributes to the literature on PQIs for home care and provides a basis for future research and practice. These selected PQIs should be applied to future research to test their applicability and validity within home care programming and outcomes. Researchers should apply these selected PQIs in future studies to evaluate their applicability and validity within home care programming and outcomes.

**Supplementary Information:**

The online version contains supplementary material available at 10.1186/s12913-024-11299-z.

## Highlights

This study aimed to identify a set of financial performance and quality indicators (PQIs) for evaluating home care in Canada. The researchers used a modified multi-phased Delphi research approach with endorsed consensus on three PQIs from the 34 reviewed. One PQI is operational, while the other two are systems-policy-focused.

This study was motivated by the need to measure and improve the financial sustainability and efficiency of home care services in Canada. It contributes to the literature on PQIs for home care and provides a basis for future research and practice.

This research is novel as it is the first to identify key cost-based PQIs through a national expert panel and modified Delphi methodology for use within a broader health system measurement framework, such as the Institute for Healthcare Improvement Quadruple Aim framework.

The indicators identified in this study may provide an essential foundation for measuring operational and health system outcomes and require further engagement at local and regional levels, further development, and conceptual application within established health system performance frameworks.

Evaluating the potential adoption and implementation of evidence-based PQIs is essential to measuring and improving home care system programming, including indicators reflective of the acceptability and potential usefulness of measures by population groups as an important future step.

**Supplementary Information:**

The online version contains supplementary material available at 10.1186/s12913-024-11299-z.

## Background

Over the last 50 years, Health Canada has recognized the need for targeted investments in community-based programming, specifically in-home care, palliative care, and mental health [1–4]. In 2017, the federal government created a new structure for provincial and territorial health transfer payments [5]. Specifically, in partnership with provinces and territories, a strategic federal investment of $11B was allocated for community-based programs, with a majority portion [$6B] allocated to home care [6, 7]. In addition to the targeted federal funding, some provinces and territories added further investments, such as in Alberta, where policymakers allocated a total of $200M, approximately 54% above the federal allocation [6, 8]. These investments support healthcare service evolution toward more community-based care [9, 10]. Still, these investments have yet to be holistically measured for impact on health outcomes, including under a healthcare measurement performance framework, such as the Institute for Health Improvement IHI Quadruple Aim [11]. Furthermore, there is a need for valid and reliable home care cost-based indicators at the national and provincial levels, and the current limitation creates a challenge for healthcare policymakers and decision-makers to determine the outcomes of these strategic funds.

Despite minimal measurement standards, healthcare systems across Canada are investing in community-based programs such as home care [9, 12]. Home care programs and services are offered across all provinces and territories in Canada, they provide scheduled care for people in their homes and communities based on their short-term (post surgery, injury or illness) and long-term (frailty, disability, living with multiple chronic conditions) [13]. According to the Canadian Institute for Healthcare Information approximately 1.4 million Canadians (3.7% of the population) received home care services in 2019 [13]. Investing in home care services in Canada are primarily provided through the provinces and reducing the use of expensive, often unnecessary, emergency department and hospital-based care, wound care, medications, and other care/personal supports for daily living.

Investing in home-based care aims to increase the quality of care, improve clients’ experiences, and reduce the use of expensive, often unnecessary, emergency department and hospital-based care [7, 13]. At the national level, these investments are unstructured, as illustrated by the average annual increase in the 2020-21 year in home and community care spending ranging from a high of 12.6% in PEI to a low of 1.9% in Manitoba [14]. It is estimated that for the fiscal year 2022, Canadian provinces will spend an average of $325 per capita on home and community care, which is less than 4% of total national healthcare estimated expenditures [15]. At the provincial and territorial levels, governments set mandates to health authorities or through their health ministries on how to structure and invest in healthcare programming, including home care service provisions.

### Context

With Canada’s growing and aging population and escalating hospital service costs, home care is critical for sustaining Canada’s publicly funded healthcare system [16]. To keep pace with population needs, home care programs require ongoing investments and outcome evaluation through reliable indicators within a healthcare measurement performance framework, including cost-based indicators. Healthcare systems in Canada and elsewhere are at crossroads of reform in response to rising economic and societal pressures [17]. Performance and Quality indicators (PQIs), also known as healthcare performance indicators are essential for measuring the effectiveness and efficiency of services in a desired direction [18]. For policymakers to gauge a level of quality and performance across the healthcare system and of components of the continuum of care such as home care programming, evidence informed measures need to be identified, validated and applied within a system-wide holistic healthcare system performance measurement framework [19].

Various organizations and health systems, such as the Ontario Local Health Integration Networks and the Canadian Medical Association, are increasingly using the Institute for Healthcare Improvement (IHI) Quadruple Aim framework as a guide to evaluate system performance in a balanced manner [20]. Additionally, with respect to healthcare research frameworks, the Canadian Institutes for Health Research (CIHR) has embraced the IHI Quadruple Aim performance model as part of its 2021–2026 strategic plan [21]. The IHI Aim quadrants provide the framework for healthcare policy makers and leaders to focus on making populations healthier, making care for individuals, making work more joyful for healthcare workers, and optimizing healthcare expenditures [22]. The optimizing healthcare expenditures quadrant focuses on eliminating waste, inefficiency, and overuse of resources in the wrong place, as well as aligning funding and incentives with value and outcomes) [22].

The Canadian Institute for Health Information (CIHI) is an organization that collects, analyzes, and disseminates health-related data and information in Canada [23]. As a mandate set by the federal government, CIHI has developed and published indicators that focus on various aspects of home care programming, such as wait times for home care access, caregiver distress scores, and inappropriate use of other systems due to the potential lack of home care services (acute and long-term care settings) [24]. These indicators help inform outcomes and performance for some quadrants of the IHI quadruple Aim, such as the quality and effectiveness of home care services (clinical outcomes) and the experiences of patients and families. No cost-based home care specific PQIs have been published by CIHI [25].

### Problem and gap

Despite the importance of understanding how specific investments change health system outcomes, there is yet to be a standard set of cost-based PQIs and related methodology for home care in Canada. This lack of standardization makes it difficult to assess and compare the quality and performance of home care services across different jurisdictions. Cost, efficiency, value for money and/or sustainability indicators are yet to be developed for home care measurement and performance at provincial and territorial levels. Due to this knowledge gap, a core set of cost-based indicators that complement an overall performance management framework needs to be identified to detect changes in home care PQIs based on investment. For example, outcome-based measures that focus on system utilization as part of cost expenditure data are necessary to show the impact of the $6B federal investments and the additional provincial home care investment, such as those making up the $200M allocations in the province of Alberta [5, 26, 27].

Federal and provincial policymakers need tools to understand these investments’ overall value to communicate the impacts of investment decisions on population outcomes. This study aims to identify a standard set of priority cost-based PQIs from the pre-identified list of 34 specific to home care programming at both system strategy and operational lenses utilizing the modified Delphi method through a national expert panel.

## Methods

### Scope of this overall research project

To understand the PQI landscape for home care globally, our team of researchers recently conducted a scoping review to identify existing PQIs for home care [28]. The scoping review methods included broad inclusion criteria to allow for any reference of a potential, conceptual or developed PQI from various sources (including but not limited to journal articles, government publications, and health authority publications) [28]. The scoping review identified 829 unique PQIs. Based on the IHI Quadruple Aim quadrants, 661 unique measures were identified as Clinical Outcome, 35 as Healthcare Provider Satisfaction, 99 as Patient Experience, and 34 in the Financial/Sustainability quadrants. Based on the broad inclusion criteria, this scoping review further recommended future research to identify core cost-based PQIs that can be conceptually applied within a healthcare measurement performance framework such as the IHI quadruple aim for home care in Canada [28].

### Study design, protocol development and ethics

Many panel techniques have been developed and used as the gold standard in indicator selection; the Delphi and Nominal Group techniques are two types of consensus methods [29–31]. Modified Delphi techniques, such as the one applied to this research, share elements of traditional Delphis and Nominal Group techniques. The modified Delphi method provides an empirical evidence-based foundation through indicators selection processes [31, 32]. These processes support satisfying selection domains to help maximize the effectiveness of developed indicators to meet the defined scope and overall quality healthcare policymakers and system executives are seeking [31, 32].

Several successful examples of modified Delphi studies have been based on community healthcare programs in Canada. For instance, a two-round modified Delphi study was undertaken with participants with extensive experience planning, implementing and evaluating community health worker programs [33]. Another example adapted a modified Delphi approach to select criteria from identified indicators and represents the initial step in the indicator development process. This study ultimately led to the creation of a final proposed set of 27 injury-related indicators specific to First Nations and Inuit children and youth. These indicators can be utilized to enhance injury prevention efforts within Canada’s Indigenous communities [34].

Various panel-driven processes across Canada have been established as best practices for indicator development. CIHI has a four-step panel-driven process that includes expert engagement within each step via panels and/or external auditing of calculations and analytic approaches [35]. The Health Quality Council of Alberta and Health Quality Ontario (HQO) models for indicator development are also foundationally structured through panel-driven approaches that focus on developing indicators that meet the consensus needs of policymakers and healthcare executives [36–38]. The modified Delphi method is a consensus strategy that, through a multi-staged approach, uses a literature review, the feedback/scoring/rankings/ratings the judgment and re-rating from experts within a field to reach an agreement [39]. It is useful when evidence is limited and relies on the collective intelligence of group members who reflect the diverse expertise of multiple individuals with in-depth knowledge on the subject discussed [39]. The method involves several stages, including developing a questionnaire, multiple rounds of feedback and refinement, and establishing consensus [40].

Guided by the CIHI and HCO approaches to indicator development, our modified Delphi research approach involved gathering feedback from an expert panel of healthcare professionals with expertise in home care services. The panel once established, was tasked through surveys and engagement sessions to score and rank individual indicators on set criteria through each progressive survey. This modified Delphi study was to identify a core set of cost-based quality and performance indicators for evaluating home care in Canada. This study was approved by the institutional ethics board [REB21-1192].

### Delphi panel recruitment and selection

Both purposive and a snowball method was employed in this study to identify potential expert panel members who can provide guidance at a health system level across various provinces, health systems, and professional experiences. Purposive sampling includes researchers intentionally selecting participants based on specific characteristics or expertise for modified Delphi panels helping identify experts who can contribute valuable insights [41, 42]. The snowball technique of requesting identified potential panelists to cascade to other potential panellists is commonly used to recruit participants for research studies through [43]. Purposive sampling complemented with snowball sampling has been used successfully in other healthcare focused modified-Delphi research [44]. This method has also been successfully utilized in research to identify rare/small population groups [45].

Experts for the panel were defined as those with extensive experience professional or academically within home care programs across Canada. Based on the estimated challenges in acquiring a sufficient panel size due to the impacts of experts being available during the active COVID-19 pandemic, we did not outline further specific characteristics of prospective panellists, such as a minimum number of years of experience or several relevant publications. We accepted those invited through the snowball method to participate who expressed interest in the panel. The snowball technique began in July 2022 through direct requests to members of a previously established national community of practice committee specific to the continuing care sector, including publicly funded home care operations and strategy. Members of this committee were invited via e-mail to both consider participation as expert panel members and to cascade the invitation across their professional networks as they deemed appropriate. We aimed to recruit 12 expert panel members representative of multiple healthcare jurisdictions and provinces across Canada. Once identified to the researcher team, we informed the potential panellist of the study objectives, provided the study background, and were invited via e-mail to participate in the panel to review, score, and identify home care indicators through a modified Delphi online survey process. Informed consent was obtained when the potential expert panel member expressed willingness to participate.

### Modified-Delphi process

**Fig. 1:**
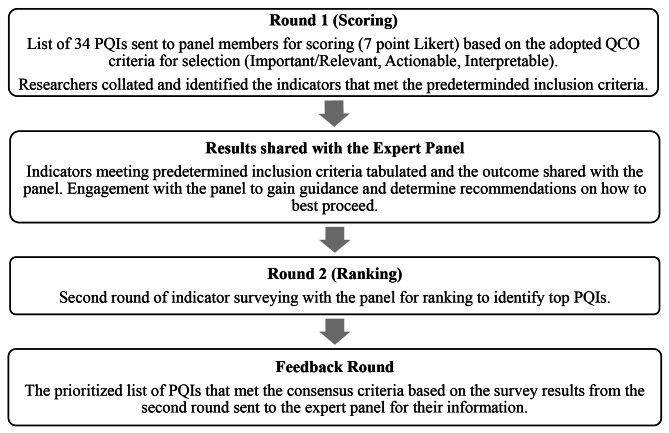
Modified Delphi process

A survey poll was utilized to identify the best dates and times to meet with the expert panel member to support their orientation. Due to the intensive activities supporting the response to the COVID-19 pandemic, the research team was challenged to identify availability for the expert panel to meet. In an effort to ensure optimal participation, each panel member was provided with an opportunity, based on their availability, to participate in one of two orientation sessions held via Zoom software (version 5.12.0) on October 7, 2022, that outlined the purpose, methods and processes they would experience [46]. The Zoom orientation sessions were recorded and shared with each panel member for future reference. The methods (Fig. 1) outlined that two online survey activities would be conducted whereby panelists would rate indicators in a first survey step and then discuss the results and re-rate in a second survey step. A stipend of $50 per activity was offered to each panel member participating in each survey and the Zoom kick-off introductory meeting. Study data were collected and managed using REDCap electronic data capture tools and downloaded to Microsoft Excel 2016 (version 16.0.5408.1001) with REDCap results stored on a secure University of Calgary network drive [47–49].

### Round 1

Each expert panel member was e-mailed a link to the REDCap survey after the second orientation Zoom session concluded. The auto-generated e-mail provided each panel member with a unique survey link and participant identifier. The invitation and subsequent e-mail reminders were sent approximately a week apart until all panel members responded by either completing the survey or notification of withdrawal. The first-round survey included a portion that requested and collected demographic information about each expert panel member. Consent was considered obtained from each expert panel member through their participation in the survey. The REDCap survey was structured so that each of the 34 indicators could be scored by criteria individually by each panel member. Based on criteria adapted from the HQO as part of Ontario’s provincial PQI selection methods the panel was asked to score each indicator using a 7-point Likert scale from strongly disagree to strongly agree on three criteria: Importance, Actionable and Interpretable (Table 1) [50]. Congruent to the approach taken by Health Quality Ontario in selecting general home care indicators, the 7-point Likert scale was chosen for our survey [50]. The survey was developed for this study and not published elsewhere. Two research team members piloted the REDCap survey to ensure clarity of wording and suggested time to complete. The survey is available in the supplemental materials.

**Table 1:** Home care indicator selection criteria

Criteria for Selection	
Important/Relevant	The PQI reflects an issue that is important to the general population and relevant policy-makers in the health system.
Actionable	The indicator is likely to inform and influence public policy or funding, alter behaviour of health care providers, and/or increase general understanding by the public in order to improve quality of care and population health.
Interpretable	The indicator is clear and can be easily interpreted by a range of audiences; the results of the indicator are comparable and easy to understand, including what constituted improved performance, such as clear directionability.

Criteria and methods utilized are adapted from the Health Quality Ontario [50]

The predetermined inclusion/exclusion criteria are also adapted from the HQO efforts in that based on the survey results, PQIs meeting one of the following will be brought forward for further ranking: Criteria A: at least 10% of respondents scored the PQI as Agree or Strongly Agree overall average (Likert); or Criteria B: 100% of respondents scored the PQI in one of the three agreement domains (Somewhat Agree, Agree or Strongly Agree) [50]. If none of the scored PQIs met these criteria, the top ten would be brought forward for final ranking. These highest ranked PQIs would be discussed with the members rakning their top five. As part of the first round, expert panel members were invited to submit additional indicators for discussion and potential inclusion in subsequent rounds. The first round of surveying concluded on November 7, 2022, when the last expert panel member submitted their survey response. The survey is available within the supplemental materials.

### Round 1 analysis and discussions

During this phase, we exported survey data from REDCap into Microsoft Excel software. We reviewed and organized the information into specific tables based on demographic data and each inclusion criteria. A review of the tables was conducted to ensure accurate analyses of the responses from each of the panel members and if they met either the criteria A and B by examining the responses and the calculations embedded within and the outputs from REDCap. For criteria A, score of less than 10% of panellist scoring the indicators across the three domains did not meet the inclusion criteria. For criteria B, we visually examined each of the domains and only those where every respondent had scored at least one of the domains as at least somewhat agree were flagged as meeting the criteria. We sent the tabulated results based on predetermined inclusion criteria via e-mail to the panel, including the scoring of the PQIs. Over January and February 2023, a qualitative discussion with the panel members occurred through e-mail exchange, including dialogue on recommendations to best approach the second round of survey activity to achieve consensus. E-mail was chosen to host the discussions due to the challenges of responding to the COVID-19 pandemic and organizing a large national group for virtual meetings. Independent polling of the members’ recommendations for the consensus round was gathered as part of the dialogue. A poll was developed during this activity and not published elsewhere.

### Round 2 and feedback round

A second round of surveying with the goal of achieving consensus via ranking top indicators was developed and shared with the expert panel. We sent the panel members an e-mail containing a Microsoft Excel spreadsheet developed to allow panel members to examine all remaining indicators and identify their top indicators. Once complete, these rankings were sent to the research team for tabulation. The research team translated each response into a final grouping of indicators using Microsoft Excel spreadsheet software. Top indicators were those that had over 50% of panel members who ranked them within their top five selections. The results were shared with the panel members, including their top majority-ranked indicators.

## Results

The recruitment techniques resulted in 22 prospective panellists being invited as expert panel members. Ultimately, 13 individuals from across Canada came forward expressing interest in participating, with 12 of the 13 having completed the first modified Delphi survey and one panellist being unable to meet the commitments of this research. Unfortunately, not all provinces and territories, such as Quebec, had representatives identified. As illustrated in Table 2, the expert panel represented 11 publicly funded institutions and organizations (five health authorities, five universities, and one provincial government/ministry) and one from a non-profit organization (caregiver-focused). Most of the expert panel identified as female [67% versus 33% as male]. Every member had obtained at least a master’s level education, with six having also obtained an academic and/or medical doctorate. Additionally, four participants held Certified Health Executive designations from the Canadian College of Health Leaders.

**Table 2:** Delphi panel survey round one participants (*n* = 12)

Organization	Province	Primary area of specialty
Eastern Health	Newfoundland	Operations Leadership
Nova Scotia Health	Nova Scotia	Strategy and Policy
Nova Scotia Health	Nova Scotia	Operations Leadership
Canadian Centre for Caregiving Excellence	Ontario	Strategy and Policy
University of Waterloo	Ontario	Clinician scientist
University of Waterloo	Ontario	Research
York University	Ontario	Academic Teaching
University of Manitoba	Manitoba	Research
Shared Health Manitoba	Manitoba	Research
Dept. of Family Medicine, U of AB	Alberta	Clinical: Care of the Elderly
Alberta Health	Alberta	Strategy and Policy
Alberta Health Services	Alberta	Operations Leadership

### Results pathway

**Fig. 2:**
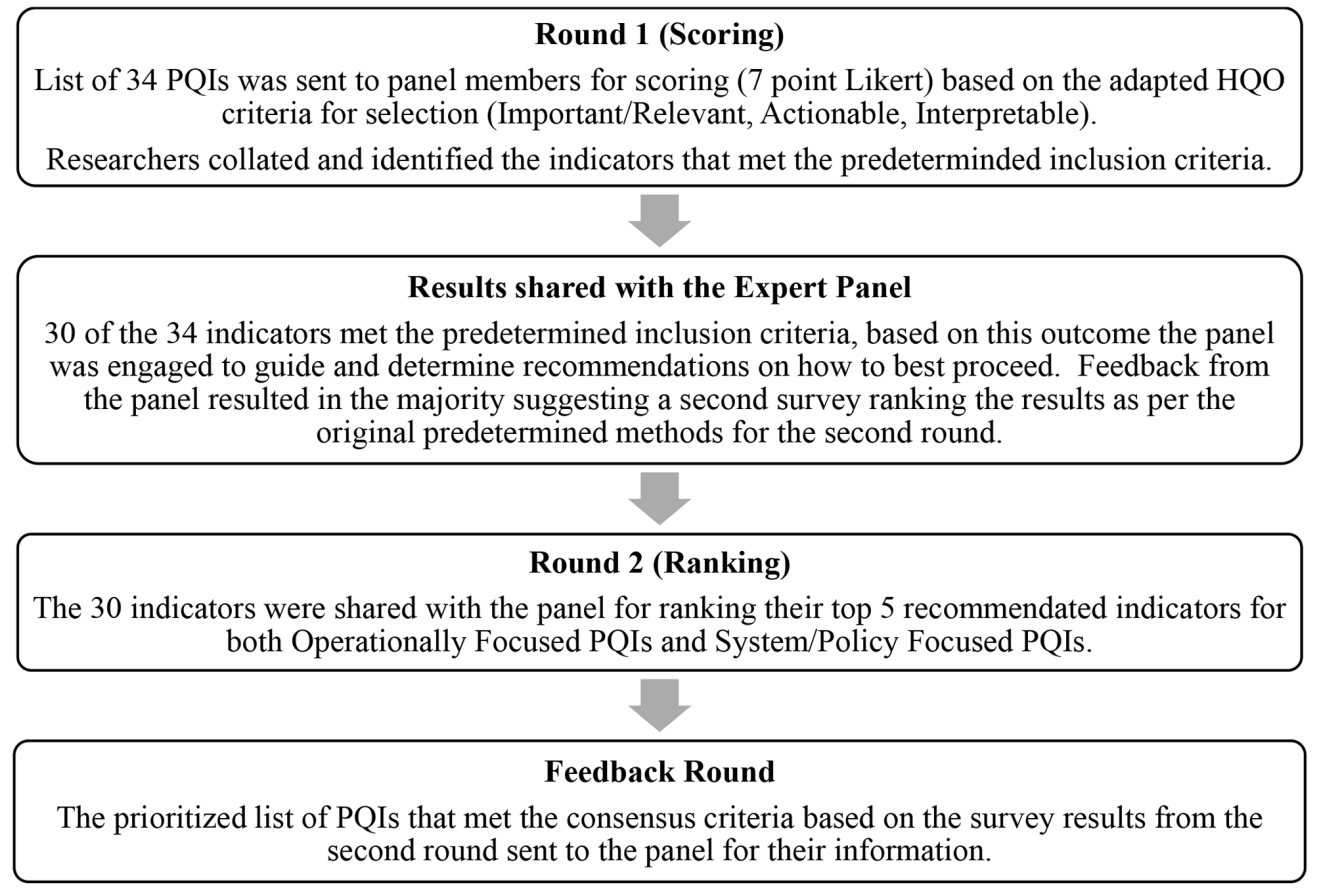
Modified Delphi process phases

The activities and results of each of the four phases of the modified Delphi processes moved in conjunction with the predetermined methods of this research (Fig. 2). Adding complexity to this research was that this was being conducted during the COVID-19 pandemic, significantly impacting the availability and responsiveness of the expert panel membership as each member was congruently working in and on the healthcare system.

### Modified-Delphi round 1 results

In Round 1 of the survey, the results identified that 30 out of 34 indicators met at least one of the two predetermined inclusion criteria and eliminated four PQIs (8, 26, 27, 33. Table 3). Four PQIs did not meet either predetermined inclusion criteria of achieving at least 10% of respondents scoring the PQI as an Agree or Highly Agree overall Likert ratings mean across all three HQO criteria or 100% of respondents scored the PQI in one of the three domains as either a Somewhat Agree, Agree and Highly Agree [50]. Indicators based on the survey that did not meet either inclusion requirement were removed from further modified Delphi rounds.

Four comments were provided in response to the section allowing panel members to suggest new indicators. Each of the comments was assessed for consideration. The comments pointed to either a general statement on measurability, were not directly related to a cost-based indicator but related to applying filters or described attributes within the current list of indicators (as suggested within the comments). None of the comments suggested new cost-based home care-specific indicators not already included within the 34. Detailed results of the first round can be found in the Appendix **A** and B tables.

**Table 3:** Round one survey results by highest to lowest inclusion scores

Indicator Name and Short Description	Predetermined inclusion/exclusion criteria	
	Criteria A: Percent of Responders that scored the PQI as Agree or Highly Agree overall Likert score (10% or greater as inclusion)	Criteria B: 100% of respondents scored the PQI in 1 of the 3 domains as either a Somewhat Agree Agree and Highly Agree as inclusion
Indicator 11 “Emergency Department and Acute service cost (mean) per home care client 1 y, 3y and 7y (all publicly funded services) per health authority and/or provincially and/or nationally”	67%	Yes
Indicator 34 “Total cost of care last 12 months of life per home care client”	58%	No
Indicator 7 “Home care office outsourced home support services provided (no, private, non-profit)”	58%	No
Indicator 1 “Funding per capita”	50%	Yes
Indicator 18 “Average cost per day per home care client”	50%	No
Indicator 2 “Home care funding as a percent of overall health care budget”	50%	Yes
Indicator 31 “Percent change in baseline vs follow up in money spent in the last month on medical care”	50%	No
Indicator 32 “Percent change in baseline versus follow up in out of pocket payments for all medical costs in the last month”	50%	No
Indicator 6 “Home care service cost (mean) per home care client 1 y, 3y and 7y per health authority and/or provincially and/or nationally”	50%	No
Indicator 10 “Population based - hospital, cost per patient (6 month)”	42%	No
Indicator 19 “Total health care costs first 30 days post discharge per home care client”	42%	No
Indicator 29 “Percent change in baseline versus follow up in financial hardship”	42%	No
Indicator 5 “Entire population - mean home care cost per patient (6 month)”	42%	No
Indicator 9 “Population based - outpatient (ambulatory, emergency department) cost per home care patient (6 month)”	42%	No
Indicator 4 “Percent expenditures per local health authority by acute, homecare, public health/mental health).”	33%	No
Indicator 12 “Community based cost (mean) per home care client 1y, 3y and 7y (all publicly funded services) per health authority and/or provincially and/or nationally”	33%	No
Indicator 14 “Population based - Total (hospital, LTC, outpatient and home care) costs per home care patient (6 month)”	33%	No
Indicator 15 “Average home care cost per patient per 3 months (total, acute, ED, home care, primary care)”	33%	No
Indicator 22 “Cost per wound treatment per home care client”	33%	No
Indicator 17 “6 month mean home care office client health service use and costs”	25%	No
Indicator 20 “Cost per patient in hospital generated costs (per home care client per duration of services/per visit). Includes costs associated with readmissions specifically”	25%	No
Indicator 21 “Assessment of the spending of a home health agency’s Post Acute Care Home Health episode relative to the spending of the national median home health agency’s Post Acute Care Home Health episodes across the same performance period”	25%	No
Indicator 23 “Annual expenditures (home care office)”	25%	No
Indicator 28 “Percent change in baseline versus follow up in patient caretaker/caregiver lost wages”	25%	No
Indicator 30 “Percent change in baseline vs follow up in lost wages”	25%	No
Indicator 3 “Difference in total care costs among like clients”	17%	No
Indicator 13 “Population based - overall community-based costs per home care patient overtime (6 month)”	17%	No
Indicator 16 “Total (mean) cost per home care client 1 y, 3y and 7y (all publicly funded services) per health authority and/or provincially and/or nationally”	17%	No
Indicator 24 “Home care service cost (mean) per home care client 1y, 3y and 7y (all publicly funded services) per home care office”	17%	No
Indicator 25 “Home care service cost (mean) per home care client 1 y, 3y and 7y per home care office”	17%	No
Indicator 26 “Mean total cost of publicly funded home care services per home care client per home care office”	8%	No
Indicator 33 “Percent change in baseline versus follow up in sale of personal belongings to pay for medical care”	8%	No
Indicator 8 “Looking at an entire population - LTC cost per home care patient (6 month)”	8%	No
Indicator 27 “Community based service cost (mean) per home care client 1 y, 3y and 7y (all publicly funded services) per home care office”	0%	No

### Results shared with expert panel

Discussions were held via e-mail with the panel on approaching the next round to achieve consensus. Complicated by the limited availability of the expert panel members, the challenge faced by the researchers and the expert panel was how to best approach consensus on a core set of indicators, as the first survey identified nearly all indicators meeting the inclusion criteria. Understanding the constraints on panel members’ ability to participate in an interactive live discussion (due to the impacts of the COVID-19 pandemic), we collectively worked with the panel to identify a feasible path forward to achieve consensus. The panel provided guidance on two options; to conduct another survey (including structure) or have the researchers suggest adjustments to the inclusion/exclusion criteria (such as to tighten the Likert inclusion score means) to potentially exclude additional indicators. Most expert panel members recommended a second survey focusing on PQI rankings. Additionally, these responses included additional guidance on how to approach the final consensus survey best.

Based on research team discussion and expert consultations, surveys were created to allow expert panel members to provide their rankings, one from an operational perspective and one from a policymaker panel members’ perspective. This approach incorporated feedback from the panel and was built to reflect both strategic/policy and clinical operations perspectives.

### Consensus process

The second survey was structured to allow the panel to rank their recommended top indicators from the remaining 30 indicators from the first round. The second round of surveying requested that the panel select up to five of the most important PQIs specific to operational needs and up to five PQIs specific to the system/policy categories. The same indicators could be selected for each category. Indicators identified by at least 50% of the expert panel participants would be selected as consensus by the expert panel. Eight of the 12 panel members responded to the second survey with one expert panel member electing to only rank specific to policy/system. Four panel members withdrew from this stage of the study due to an inability to complete the requirements within the allotted timeframe (one member retired from service, and three others gave no reason). Overall, we retained close to 67% of the original membership with an attrition rate of 33%.

### Consensus results

After the second round of the modified Delphi cycle, consensus was gained for three PQIs: one health system measure and two specific to the operational performance lens (Table 4). The health system PQI (indicator 2) was also one of only three indicators that met both inclusion criteria in the initial survey (Table 4). The “home care funding as a percent of overall health care budget” (indicator 2) received 7/8 expert panel members ranking as a top health system measure PQI. The “average cost per day per home care client” (indicator 18) and the “home care service cost (mean) per home care client 1y, 3y and 7y per health authority and/or provincially and/or nationally” (indicator 6) both received 5/7 expert panel members ranking as a top operational performance measure PQI (Table 4).

**Table 4:** Round two survey results

**Health Systems Measures** - Indicator Ranking Health Systems Measure - The benefit/impact potential the indicator will provide to health system policy makers and executive teams in supporting decisions on how to best evolve/invest in/reshape the health system to achieve overall improvements in health system outcomes.	% That Responded as “Include” *n* = 8
**“Home care funding as a percent of overall health care expenditures” [Indicator 2]** To allow visibility to the percent of spend of the overall home care programming as a portion of the overall healthcare budget. As local health authorities enhance services to the community, % spending should shift over time to increase the overall % budgeted. Additionally, this measure allows for comparability across local health authorities. Financial Components: Total publicly funded healthcare expenditures proportioned by home care programming. Source [51]	88%
**Operational Performance Measures** - Indicator Ranking Operational Performance Measure - The benefit/impact potential the indicator will provide to Operational executive teams in supporting decisions on how to best evolve/invest in/reshape the health care operations to achieve overall improvements in program outcomes.	% That Responded as “Include” *n* = 7
**“Average cost per day per home care client” [Indicator 18]** Purpose of the measure: To allow for an understanding of how clients receive care based on the day of the week. Understanding that services are typically less available on weekends and holidays. Financial Components: Mean total costs for home care services per home care client per day. Source [52]	57%
**“Home care service cost [mean] per home care client 1y**, **3y and 7y per health authority and/or provincially and/or nationally” [Indicator 6]** Purpose of the measure: To understand the actual total expenditures (health authority, municipal, government, etc.) of home care clients for solely home care services (can be compared year over year and across jurisdictions) over time, including how these changed over the last 1, 3 and 7 years. Financial Components: Total home care service expenditures. Source [53]	57%

## Discussion

Guided by experts from various health systems across Canada, through our modified Delphi method, our research has identified three PQIs specific to supporting the measurement of home care programming that fit within the financial quadrant of the IHI Quadruple Aim framework. This is the first such identification of a set standard of home care-specific cost-based indicators for use within the IHI quadruple aim framework. The final list of PQIs “home care funding as a percent of overall health care budget” (indicator 2), “average cost per day per home care client” (indicator 18), and “home care service cost (mean) per home care client 1y, 3y and 7y per health authority and/or provincially and/or nationally” (indicator 6) was identified through the Delphi research processes. This research study demonstrates that utilizing a modified Delphi approach to indicator selection can effectively achieve consensus among an expert panel that comprises individuals across provinces and diverse professional backgrounds and roles.

### Why this study was needed

In Canada and across the provinces, there have been numerous attempts to boost home care funding, yet without a standard approach to monitoring home care expenditures, and these announcements are not measured nor reported upon in a meaningful way, resulting in continued variation of services, investments, and outcomes for Canadians. Canadian policymakers thus have an opportunity and an obligation in their attempts to sustain the publicly funded healthcare system by adopting a standard set of cost-based PQIs specific to home care programs. The IHI quadruple aim offers a balanced approach to measuring the outcomes of shifts and changes to the health system. It is a strategic planning and measurement framework that aligns healthcare strategies with performance. It is understood that when a balanced measurement framework is applied to healthcare, frameworks such as the IHI Quadruple Aim can be beneficial in evaluating and improving the performance of healthcare organizations [54]. Measurement frameworks have been shown to successfully provide performance benchmarking of health service capacity and service delivery, stimulate new dialogue about organizational vision and strategy, and instigate change [54]. To implement the IHI quadruple aim or other balanced scorecard frameworks, there is a need for a concise set of validated indicators within each quadrant, as the implementation of these frameworks must be tailored to the specific needs and context of each healthcare system [11, 55].

### Selected indicators

Budget allocation for healthcare expenditure (indicator 2, “home care funding as a percent of overall health care budget”) is an important indicator to showcase to policy and system leaders the percentage of the overall healthcare budget allocated to this vital segment of the system. Based on Canada’s growing and aging population and the projected impact on the entire health system, the ability to build more facility-based capacity (Hospitals and Long-term Care) will not keep up with the needs and domain of the population over the next 10–15 years [56]. Programs that help shift care from hospitals to the community are responsive to predicted (scheduled) and unpredicted care needs (urgent, injury, illness). Supporting people in their own homes is one of the pillars of sustaining a publicly funded health system. Effective home care programs can prevent inappropriate hospital visits, support earlier discharge from hospitals, delay/prevent the need for higher levels of care, such as Long Term Care, allow for high-cost hospital services to be provided in the home (palliative/end-of-life), and support client’s wishes and values of being cared for at home for as long as possible. These programs align with clients’ desires and values, promoting extended care at home and enhancing workforce satisfaction as they can better fulfill their role in supporting clients. This efficient utilization of resources could greatly improve the efficiency of healthcare system [9, 10]. These services include after-hours primary care access, virtual hospital structures, enhanced paramedic programs, community intravenous therapy, and home care professional and non-professional support [10, 12]. Indicator 2, especially for provinces that project multiple-year budgets, showcases the investments in home care (either as increasing funding or the lack of action). This indicator can be applied to those provinces and health system programs that project multiple-year budgets. This sets the tone for healthcare leaders to ensure mandates are met through allocated budgets. Policymakers should be aware of how increased home care expenditures reduce other expenditures or activities within the system [57]. Understanding and having clear visibility of the percentage of home care budget as a total of healthcare budgets and the impacts on actual emergency department and acute care expenditures together create a balanced health system performance indicator set.

The two operational PQIs selected (“average cost per day per home care client” (indicator 18), and (indicator 6) “home care service cost (mean) per home care client 1y, 3y and 7y per health authority and/or provincially and/or nationally”) focus on healthcare expenditures per home care client per day and total annual services with year-over-year and health system/provincial comparisons. Understanding expenditures linked to how clients are accessing care at a daily level is an essential operational indicator, as it allows for greater awareness of the cost per care for various client types (long-term, short-term, palliative, pediatrics) and how geography, home care office structure, care teams composition, seasonality and business hours versus after hours is structured. In terms of value for investment, there is a significant opportunity to understand home care costs and how spending coincides with larger health system expenditures, utilization, and outcomes [58, 59]. Comparing daily expenditures with acute care service/expenditure data may also reveal that expenditure differences at the home care daily level impact activity data for higher costs and more scarce resources such as emergency departments and acute care services. Additionally, comparing year-over-year expenditures per home care client will reveal new information about the allocation of provincial resources on individual home care clients and how this is changing at the operational level for front-line leaders and teams. Notably, by having these comparisons across the health system and provinces and compared year-over-year, such as the last 1st, 3rd and 7th year, multi-year trends associated with election cycles and other external influences may be witnessed.

These three core indicators compare to the other 31 in various ways. The majority of indicators not selected are related to costs per client in theme, such as cost per home care client for community-based services or hospital, emergency, and/or by home care office. The cost per client by type, location or service are subsets or variants of the two core operational performance measures identified through the panel that focus on overall cost per client. Notably, no other indicators among the 33 were similar to the selected core indicator of home care funding as a percent of the overall healthcare budget. Other indicators not selected but important and requiring a further understanding of how these should be considered are those related to patient and caregiver costs associated with home care. Some provinces have a co-pay model that can further burden those needing publicly funded homecare services. As identified by the BC Seniors Advocate, these added costs can be a barrier to accessing services, negatively impact overall citizen outcomes, and drive higher levels of more costly care [60].

Conceptually, with these three indicators applied to the IHI Quadruple Aim, policymakers are able to see and understand the impacts of investment and funding decisions across the health system and all quadrants. For example, home care is a service that provides care for a significant segment of the population; home care service structures, funding, and availability have a direct impact on population outcomes. In considering how these relate to the other quadrants, it can be hypothesized that when home care program investments are reduced compared to population needs, the effect on the healthcare system outcomes quadrant is increased use of higher levels of health services such as acute care and facility-based care (long-term care) and at a higher overall system cost. Experiences with healthcare for clients, families, and community will shift, and if home care becomes too scarce or does not serve necessary unmet needs, we could witness specific scores from annual assessment tools, such as caregiver burnout being negatively impacted. Furthermore, home care service funding decreases may impact the responsiveness of the programs, such as upon health status changes and/or the ability to support caregivers, ultimately leading to negative impacts on client experience. Lastly, we know that there is an increased moral distress experienced by healthcare workers when they do not have adequate resources to support patients and families. Conceptually, we could observe satisfaction scores with the health system, with the employer, and as professionals decrease.

There is also a need to reassess the use of the IHI Quadruple Aim and consider shifting towards the IHI Quintuple Aim for future studies. As an outcome of the COVID-19 pandemic, the IHI Quadruple Aim has evolved, with a fifth Aim proposed by the IHI [61, 62]. The Quintuple Aim includes the additional dimension of advancing health equity [61, 62]. When measuring home care health system performance, this fifth Aim emphasizes equitable access, outcomes, and experiences for all individuals, regardless of their background or circumstances. It ensures that healthcare delivery considers the unique needs of diverse populations and strives for fairness in resource allocation and service provision. Conceptually, one approach to applying the principle of the fifth Aim under the current set of identified core cost-based indicators could be by applying specific filters that focus on marginalized populations. Examining cost-based indicators comparing those with lower social determinates of health deprivation scores in health system evaluation frameworks, we can understand further how investments in home care are dispersed and if inequities exist across various groups.

### Data sources and applicability

Beyond the specific indicators identified by the expert panel, our findings highlight some important challenges and opportunities for performance measurement initiatives in terms of application, data sources and feasibility of implementation. Health system performance measurement is only as effective as the data that supports it. In Canada, financial data (cost or budget) and reports specific to home care expenditures are not standardized or readily available across health systems (or even within health systems at times). For example, in Alberta, there is a greater degree of reporting as the Ministry of Health publishes multi-year budgets for home care each year, and Alberta Health Services (provincial health authority) reports actual home care expenditures annually. At the same time, in British Columbia, no provincial budget is published specific to home care services; each health authority provides published annual budgets that do not include details on home care services and home care expenditures are not reported publicly. With this identified set of core cost-based indicators, various healthcare organizational leaders and policymakers can begin to consider these in how financial reporting (costs and budgets) structures are developed. Our findings reinforce the fundamental role of context and values within health system stewardship and the importance of aligning health policy with broader agendas.

## Validity, weaknesses, limitations

There are limitations, critical reflections and areas for future discussions and efforts that need to be considered when examining our results. It is understood that modified-Delphi studies’ validity depends on rigorous processes and ongoing refinement [63]. While consensus-based results provide valuable insights, further testing and development are often necessary to ensure practicality, which is a common next step [64]. For our study, there is a need to build upon these system quality and performance home care indicators under scientific methods, including conceptually applying these at the national and provincial levels as part of further research. As these selected PQIs were not assessed by the expert panel for appropriateness or feasibility in this study, there is a need for further evaluation of the indicators in terms of feasibility and usefulness as a next important step. Not publishing the criteria prior to the launch of the study impacts the validity of the study approach and is a weakness that should be considered by policymakers and operational leaders when considering the application of the identified three core cost-based PQIs.

The expert panel was not selected to require the knowledge of how to develop these indicators, apply these indicators within complex systems, or assess feasibility. The validity and reliability of these indicators have not been examined in this research. As part of this study’s design, these indicators were identified using importance/relevance, action-ability and interpretability as selection criteria, along with a ranking exercise. The indicators reviewed by the expert panel were identified from 16 sources. The three identified PQIs were referenced once, each from separate manuscripts. This work provides a foundation to test these indicators to understand their feasibility, applicability, and appropriateness within a healthcare system within Canada.

The decision to include a second modified Delphi survey based on the expert panel’s guidance to rank the remaining PQIs is an augmentation from typical modified Delphi processes as was chosen to support the feasibility of the study being completed. It is understood that modifying the method in partnership with the expert panel is a weakness of the study design and the overall quality of this study. Even though panellists were engaged in deciding how consensus should be achieved, best practice standards are that pre-published criteria and methods provide high validity to the research outcomes. Mitigating these weaknesses is the composition of the expert profile of the panel, being those with deep experiences and education specific to home care services and strategy, adding to improved construct and content validity. Additionally, due to the expert panel representation and the broad description of each of the three indicators, our results are generalizable in nature, as the findings conceptually could be applied to most health systems [65, 66].

Due to the impacts of the COVID-19 pandemic at the time, recruitment and retention of panellists was a challenge, impacting the study’s reliability. Even though we recruited representatives from many areas across Canada, we did not successfully acquire representation from each province (such as Quebec). Additionally, eight experts participated in the final round, in which one expert only participated in one of the two ranking surveys. It is understood that commonly the number of expert panel participants ranges from eight to 20 [67]. Even though a 33% loss ratio is not uncommon in modified-Delphi studies within healthcare, and considering having retained 66% of participants as they were also pre-occupied with responding actively to the impacts of the COVID-19 pandemic, the results reached in the final ranking round may be biased in favour of the experts that remained. Even though purposive sampling and snowball sampling are common methods for identifying and recruiting expert panel members, the process can create potential biases, such as conformity pressure [68]. A further limitation is that the snowball approach may have led those invited to consider participating only recommending others they agree with, reinforcing any bias in the initial sample [44]. We believe that we minimized these limitations by having a panel comprised of various experts from across provinces, institutions, and health systems who had a wide range of academic training and professional experiences.

### Recommendations and future directions

The scoping review that informed this research indicated home care performance systems are not mature in their development and are not organized in a way that allows for a health system to measure impacts using a balanced health system performance and measurement framework [28]. Within this research, while the selected indicators are not yet ready for implementation as they have been presented here, Canadian health regions interested in improving their use of contextually relevant cost-based performance indicators may find these relevant.

A significant challenge for health systems is the systematic collection of data, information and its ability to effectively and adequately apply it to build out measures. A data analytics architecture that includes data scientists with a robust understanding of system structures is required, especially in more challenging, under-resourced systems. There is a lack of a system-focused home care evaluation framework with balanced measures across all health system performance framework quadrants. Concomitant development and application of a robust health system performance framework such as the IHI Quadruple Aim with other indicators comprising of the other quadrants will further provide comprehensiveness and relevance to these financially focused indicators while minimizing indicator redundancy.

Cost-based indicators can drive policy changes in terms of budget allocations, which can, in turn, impact overall the cost of healthcare services shifts across sectors over time [69]. CIHI publishes two key indicators annually, one specifically set to report on overall healthcare expenditures per person across Canada and the other being the cost of a standard hospital stay [70]. CIHI states that Canadians want to know if their health system is sustainable and provides good value for money [70]. Health spending represents, on average, around 40% of all program spending by provincial and territorial governments in Canada, with policymakers and governments looking at ways to deliver health services more efficiently [25, 70]. Compared to the current CIHI indicators, the three indicators identified through this research could add important and complementary information for policymakers. For example, home care funding as a percent of overall healthcare expenditures compared to total healthcare costs per citizen by province could illuminate how funding allocation impacts overall healthcare expenditures.

In order to prepare these indicators for use, further refinement and development are required in partnership with health system policy and operational leaders. Secondly, the indicators require testing for validity. An essential next step to this research will be the application and proof of concept of these selected indicators within a health system in Canada. There are no known home care cost-based indicators published by other provinces or territories in Canada. We understand that the importance of our indicators for policymakers is to have our research lead to the creation of new tools that provide measurement within health systems. For example, this could conceptually occur through the application of these indicators within a province such as Alberta, as numerous reports and strategies have been published in the province, such as the 2002 Seniors Strategy [71], the 2008 Alberta Health Continuing Care Strategy [72], the Alberta Health 2010 Hollander Report on home care [16], and Alberta Health Services 2020–2023 Health and Business Plan [12]. These strategy documents contain consistent messaging pointing to the need to shift care to the community through home care programming as a means of system sustainability.

Nevertheless, cost-based measurement or performance indicators still need to be created [12, 16, 71, 72]. Opportunity exists in applying these indicators to the outcomes of Alberta’s 2017 provincial government announcements of $200M in new investments for home and community care, as the majority of the investments targeted to increase home care services for all Albertans [27]. At the time of these investment announcements, robust economic evaluations, indicators or methods were not described by policymakers, and to date, they have yet to be published [28].

## Conclusion

Our scoping review identified the need for a common application of clearly defined universally accepted PQIs for evaluating home care service delivery and outcomes utilizing the IHI Quadruple Aim. This study identified three cost-focused PQIs that are important and relevant to the publicly funded Canadian health system. This novel research is the first where a national expert panel and the modified Delphi methods where key financial PQIs for use within a broader balanced health system measurement framework. The core indicators identified in this study may provide an essential foundation for this work but require further engagement at local and regional levels as well as the further development and conceptual application within region-specific health system performance frameworks.

Recognizing that successful health system performance measurement strategies require centralization and oversight; future efforts are recommended to focus on the financial quadrant of the IHI Quadruple Aim framework to support a balanced approach. There is a need for ongoing research in this area leading to the development of PQIs supported by expenditure data systems with appropriate ownership and governance including research to better understand how to effectively incorporate newly developed indicators into performance-improvement initiatives; and, within current health system evaluation and performance management frameworks. Lastly, there is a need for further evaluating the potential adoption and implementation of evidence-based PQIs is essential to measuring and improving home care system programming including indicators reflective of acceptability and potential usefulness of measures by population groups as an important future step.

### Electronic supplementary material

Below is the link to the electronic supplementary material.

Supplementary Material 1

Supplementary Material 2

Supplementary Material 3

## Data Availability

Data is included in this published article via the results tables. Upon request, authors are prepared to send relevant documentation or data to verify the validity of the results presented. This could be in the form of anonymized data, samples, or records. No participant identifiable data will be shared.
