# Supplementary material for: Identifying cost-based quality and performance indicators for home care: a modified delphi method study
Source: BMC Health Serv Res. 2024 Jul 24;24:835. doi: 10.1186/s12913-024-11299-z (PMC11270956; doi:10.1186/s12913-024-11299-z)
Supplement: Supplementary file 1 — Supplementary Material 1 [file 12913_2024_11299_MOESM1_ESM.docx]

**Appendix A – 34 Indicators by Health Systems Measures Rank and Survey 1 Scoring Results**

| **Health Systems Measures** - Indicator Ranking  Health Systems Measure - The benefit/impact potential the indicator will provide to health system policy makers and executive teams in supporting decisions on how to best evolve/invest in/reshape the health system to achieve overall improvements in health system outcomes. | % "Include" n=8 | **Rating From Round 1**  Survey 1 Results (higher 8 = higher importance rating) |
| --- | --- | --- |
| **"Home care funding as a percent of overall health care budget"**  To allow visibility to the % of spend of the overall home care programming as a portion of overall healthcare budget. As local health authorities enhance services to community, % spend should shift over time to increasing the overall % budgeted. Additionally, this measure allows for comparability across local health authorities.   Financial Components: Total publicly funded healthcare expenditures proportioned by home care programming. | 88% | 6 |
| **"Emergency Department and Acute service cost (mean) per home care client 1 y, 3y and 7y (all publicly funded services) per health authority and/or provincially and/or nationally"**  Purpose of the measure: To understand the actual expenditures (health authority, municipal, government, etc.) of home care clients for acute based services (can be compared year over year and across jurisdictions) over time, how have these changed over the last 1, 3 and 7 years.   Financial Components: Total publicly funded expenditures including pharmacy costs. | 50% | 8 |
| **"Population based - Total (hospital, LTC, outpatient and home care) costs per home care patient (6 month)"**   Purpose of the measure: To understanding the actual total expenditures (health authority, municipal, government, etc.) of home care clients on the system (can be compared year over year and across jurisdictions). Can be at regional geography level, provincial and/or nationally. Actual expenditures and growth year over year is an indicator showcasing government/health system commitments or lack of in terms of shifting care to the community. Additionally, this indicator allows for visibility of total cost of care for home care clients within the system.   Financial Components: Total publicly funded healthcare expenditures over a 6 month period per client. | 50% | 4 |
| **"Home care service cost (mean) per home care client 1y, 3y and 7y per health authority and/or provincially and/or nationally"**  Purpose of the measure: To understand the actual total expenditures (health authority, municipal, government, etc.) of home care clients for solely home care services (can be compared year over year and across jurisdictions) over time, including how these changed over the last 1, 3 and 7 years.   Financial Components: Total home care service expenditures. | 38% | 6 |
| **"Percent expenditures per local health authority by acute, homecare, public health/mental health)"**  To allow for visibility to the % of spend on home care vs acute services vs community broken down by service areas (public health and mental health). As local health authorities enhance services to community, % spend should shift over time. Additionally, this measure allows for comparability across local health authorities.   Financial Components: Total publicly funded healthcare expenditures proportioned by areas within the continuum of care. Pharmacy, DI, physician billings may be included. | 38% | 5 |
| **"Home Care funding per capita"**   To allow for an understanding of budget allocated to home care services (for comparisons such as year over year and across jurisdictions). Can be at the health authority level, provincial and/or nationally. Funding and funding growth year over year is an indicator showcasing government/health system commitments or lack of in terms of shifting care to the community.   Financial Components: Funding is directly tied to home care programming. Excludes indirect funding (pharmacy for example). | 38% | 6 |
| **"Difference in total care costs among like clients"**  To compare home care clients to non home care clients or like home care clients from various jurisdictions in their differences in total healthcare expenditures. Based on overall service design and service utilization pathways home care clients utilize higher levels of services (more costly) at a lesser rate than like individuals who are not home care clients, or home care clients who have less home care service programming available.   Financial Components: Total publicly funded healthcare expenditures proportioned by areas within the continuum of care. Pharmacy, DI, physician billings may be included. | 38% | 4 |
| **"Total cost of care last 12 months of life per home care client"**   Purpose of the measure: To allow for visibility of acute and ED usage, higher costs indicate higher levels of care usage (meaning palliative home care was not optimally effective). Local program home care palliative client expenditures compared to the average (regional, provincial, national) in the same time period.   Financial Components: Total publicly funded expenditures including pharmacy costs for the last year of life per home care client. | 25% | 7 |
| **"Population based - outpatient (ambulatory, emergency department) cost per home care patient (6 month)"**   Purpose of the measure: To allow for comparability of home care client expenditures and how these correlate to outpatient expenditures and inpatient expenditures. Home care cost per patient (health authority, municipal, government, etc.). Financial Components: Funding directly tied to outpatient/ambulatory programming such as emergency department visits, diagnostics. | 25% | 5 |
| **"Average home care cost per patient per 3 months (total, acute, ED, home care, primary care)"** Purpose of the measure: To understand the actual total expenditures (health authority, municipal, government, etc.) of home care clients on the system (can be compared year over year and across jurisdictions). Can be at regional geography level, provincial and/or nationally. Actual expenditures and growth year over year is an indicator showcasing government/health system commitments or lack of in terms of shifting care to the community. Additionally, this indicator allows for visibility of total cost of care for home care clients within the system.   Financial Components: Total publicly funded expenditures including pharmacy costs. | 25% | 4 |
| **"Percent change in baseline versus follow up in financial hardship"**   Purpose of the measure: To allow for visibility of the shifts in self reported financial hardship of the patient. Patient reported financial hardship measure as per the interRAI assessment provides information on the ability of home care to provide a full basket of services and supports.   Financial Components: home care client self reported estimated baseline and follow up financial hardship. | 25% | 5 |
| **"Percent change in baseline versus follow up in patient caretaker/caregiver lost wages"**  Purpose of the measure: To allow for an understanding of home care patient's caregiver financial burden. A significant component of home care services is the provision of services that act as caregiver supports and caregiver respite. Caregivers provide a significant amount of time and energy to support loved ones, this measure quantifies this support through lost wages.   Financial Components: Home care client self reported estimated baseline and follow up wages. | 13% | 3 |
| **"Home care office outsourced home support services provided (no, private, non-profit)"**  Purpose of the measure: To understand the composition of vendor provided services as part of overall home care programming and allowing to compare to other components of the IHI quadruple aim. Example, differences in health authorities who outsource a higher % of home care services on reduced preventable ED visits or shorter acute LOS.   Financial Components: Understand the % home care of expenditures outsourced to vendors of overall service expenditures. Can be filtered by corporation structure by vendor (private and non-profit). | 13% | 7 |
| **"Entire population - mean home care cost per patient (6 month)"**  To allow for visibility of actual total expenditure per home care client (health authority, municipal, government, etc.). Expenditures are mostly tied to services provided, expenditures vary by jurisdiction, health authority and provide. Understanding expenditure variation allows for more understanding of inequities in service delivery.   Financial Components: Funding directly tied to home care programming. Excludes indirect funding (pharmacy for example). | 13% | 5 |
| **"Cost per patient in hospital generated costs (per home care client per duration of services/per visit). Includes costs associated with readmissions specifically"**   Purpose of the measure: To allow for an understanding of intensity of acute care service provision by home care client. Those clients with more robust and responsive home care services may have reduced lengths of stay resulting in decreased overall expenditures per acute visit.   Financial Components: Funding directly tied to inpatient programming. Includes all inpatient acute care costs (pharmacy, physician, professional, non-professional). | 13% | 3 |
| **"Average cost per day per home care client"**  Purpose of the measure: To allow for an understanding of how clients receive care based on day of the week. Understanding that services are typically less available on weekends and holidays.   Financial Components: Mean total costs for home care services per home care client per day. | 13% | 6 |
| **"Percent change in baseline vs follow up in money spent in the last month on medical care"**   Purpose of the measure: To allow for an understanding of patient self-reported financial expenditures on healthcare. Not all health care services are publicly funded, this measure illustrates the amount of out of pocket costs per home care client compared by office, health authority, and province.   Financial Components: Home care client self reported estimated baseline and follow up in personal money (out of pocket) spent on medical care. | 13% | 6 |
| **"Total health care costs first 30 days post discharge per home care client"**   Purpose of the measure: To allow for visibility of healthcare usage for the first 30 days post discharge, higher costs may indicate higher levels of care usage (meaning home care was not optimally effective).   Financial Components: Total home care service expenditures per client for the first 30 days post discharge from acute care. | 0% | 5 |
| **"Total (mean) cost per home care client 1 y, 3y and 7y (all publicly funded services) per health authority and/or provincially and/or nationally"**   Purpose of the measure: To understand the actual total expenditures (health authority, municipal, government, etc.) of home care clients on the system (can be compared year over year and across jurisdictions). Can be at regional geography level, provincial and/or nationally. Actual expenditures and growth year over year is an indicator showcasing government/health system commitments or lack of in terms of shifting care to the community. Additionally, this indicator allows for visibility of total cost of care for home care clients within the system.   Financial Components: Total publicly funded expenditures including pharmacy costs. | 0% | 2 |
| **"Population based - hospital, cost per patient (6 month)"**  Purpose of the measure: To allow for comparability of home care client expenditures and how these correlate to outpatient expenditures and inpatient expenditures. Home care cost per patient (health authority, municipal, government, etc.).   Financial Components: Expenditures directly tied to inpatient programming. Includes all inpatient acute care costs (pharmacy, physician, professional, non-professional). | 0% | 5 |
| **"Community based cost (mean) per home care client 1y, 3y and 7y (all publicly funded services) per health authority and/or provincially and/or nationally"**   Purpose of the measure: To understanding the actual total expenditures (health authority, municipal, government, etc.) of home care clients for community based services (can be compared year over year and across jurisdictions) over time, how have these changed over the last 1, 3 and 7 years.   Financial Components: Total publicly funded healthcare expenditures proportioned by areas within the continuum of care. Pharmacy, DI, physician billings may be included. | 0% | 4 |
| **"Annual expenditures (home care office)"**   Purpose of the measure: To allow for an understanding of actual annual expenditures for home care programming by office. Example, for visibility in growth year over year.   Financial Components: Funding directly tied to home care programming. Excludes indirect funding (pharmacy for example). | 0% | 3 |
| **"Population based - overall community based costs per home care patient overtime (6 month)"**   Purpose of the measure: To allow for comparability of home care expenditures and how these correlate to outpatient expenditures and inpatient expenditures. Home care cost per patient (health authority, municipal, government, etc.).   Financial Components: Funding directly tied to community based programming. Includes all community services (pharmacy, physician, professional, non-professional). Not home care program direct expenses. | 0% | 2 |
| **"Percent change in baseline versus follow up in out of pocket payments for all medical costs in the last month"**  Purpose of the measure: To allow for visibility of patient self-reported financial expenditures on healthcare. Not all health care services are publicly funded, this measure illustrates the amount of out of pocket costs per home care client compared by office, health authority, and province by month.   Financial Components: home care client self reported estimated baseline and follow up in personal money (out of pocket) spent on medical care in the last month. | 0% | 6 |
| **"6 month mean home care office client health service use and costs"**   Purpose of the measure: To understand actual 6 month expenditures for home care programming and home care use by office. To allow for visibility in growth in both usage and costs to evaluate if services per client are increasing or decreased based on expenditures.   Financial Components: Total home care service expenditures per home care service. | 0% | 3 |
| **"Assessment of the spending of a home health agency's Post Acute Care Home Health episodes relative to the spending of the national median home health agency's Post Acute Care Home Health episodes across the same performance period"**   Purpose of the measure: To allow for visibility of local program home care client expenditures 30 days total post acute care discharge compared to the average (regional, provincial, national) in the same time period. To allow for visibility of acute and ED usage for the first 30 days post discharge, higher costs indicate higher levels of care usage (meaning home care was not optimally effective).   Financial Components: Total home care service expenditures per client for the first 30 days post discharge from acute care. | 0% | 3 |
| **"Home care service cost (mean) per home care client 1y, 3y and 7y (all publicly funded services) per home care office"**   Purpose of the measure: To understand the actual total expenditures by home care office of home care clients for all publicly funded services over time, how have these changed over the last 1, 3 and 7 years.   Financial Components: Funding directly tied to home care clients service utilization. Excludes indirect expenditures (pharmacy for example). | 0% | 2 |
| **"Cost per wound treatment per home care client"**   Purpose of the measure: To allow for an understanding of wound care program operational costs. Wound care can occur in a client's home or in a community ambulatory settings, cost per client allows for visibility of efficiencies of home care programs.   Financial Components: Total cost to treat wounds (home care, medications, supplies, professional). | 0% | 4 |
| **"Percent change in baseline vs follow up in lost wages"**   Purpose of the measure: To allow for an understanding of financial hardship for home care patients. A significant component of home care services is the provision of services that act as home supports including caregiver respite. This measure quantifies this support through lost wages of the home care client.   Financial Components: Home care client self reported estimated baseline and follow up wages. | 0% | 3 |
| **"Home care service cost (mean) per home care client 1 y, 3y and 7y per home care office"**  Purpose of the measure: To understand the service costs by home care office of home care clients for all publicly funded home care specific services over time, how have these changed over the last 1, 3 and 7 years.   Financial Components: Funding directly tied to home care services. Excludes indirect funding (pharmacy for example). | 0% | 2 |

**Appendix B – 34 Indicators by Operational Performance Measures Rank and Survey 1 Scoring Results**

| **Operational Performance Measures** - Indicator Ranking Operational Performance Measure - The benefit/impact potential the indicator will provide to Operational executive teams in supporting decisions on how to best evolve/invest in/reshape the health care operations to achieve overall improvements in program outcomes. | % "Include" n=7 | **Rating From Round 1**  Survey 1 Results (higher 8 = higher importance rating) |
| --- | --- | --- |
| **"Average cost per day per home care client"**  Purpose of the measure: To allow for an understanding of how clients receive care based on day of the week. Understanding that services are typically less available on weekends and holidays.   Financial Components: Mean total costs for home care services per home care client per day. | 57% | 6 |
| **"Home care service cost (mean) per home care client 1y, 3y and 7y per health authority and/or provincially and/or nationally"**  Purpose of the measure: To understand the actual total expenditures (health authority, municipal, government, etc.) of home care clients for solely home care services (can be compared year over year and across jurisdictions) over time, including how these changed over the last 1, 3 and 7 years.   Financial Components: Total home care service expenditures. | 57% | 6 |
| **"6 month mean home care office client health service use and costs"**   Purpose of the measure: To understand actual 6 month expenditures for home care programming and home care use by office. To allow for visibility in growth in both usage and costs to evaluate if services per client are increasing or decreased based on expenditures.   Financial Components: Total home care service expenditures per home care service. | 43% | 3 |
| **"Home Care funding per capita"**   To allow for an understanding of budget allocated to home care services (for comparisons such as year over year and across jurisdictions). Can be at the health authority level, provincial and/or nationally. Funding and funding growth year over year is an indicator showcasing government/health system commitments or lack of in terms of shifting care to the community.   Financial Components: Funding is directly tied to home care programming. Excludes indirect funding (pharmacy for example). | 43% | 6 |
| **"Home care office outsourced home support services provided (no, private, non-profit)"**  Purpose of the measure: To understand the composition of vendor provided services as part of overall home care programming and allowing to compare to other components of the IHI quadruple aim. Example, differences in health authorities who outsource a higher % of home care services on reduced preventable ED visits or shorter acute LOS.   Financial Components: Understand the % home care of expenditures outsourced to vendors of overall service expenditures. Can be filtered by corporation structure by vendor (private and non-profit). | 43% | 7 |
| **"Home care funding as a percent of overall health care budget"**  To allow visibility to the % of spend of the overall home care programming as a portion of overall healthcare budget. As local health authorities enhance services to community, % spend should shift over time to increasing the overall % budgeted. Additionally, this measure allows for comparability across local health authorities.   Financial Components: Total publicly funded healthcare expenditures proportioned by home care programming. | 43% | 6 |
| **"Total cost of care last 12 months of life per home care client"**   Purpose of the measure: To allow for visibility of acute and ED usage, higher costs indicate higher levels of care usage (meaning palliative home care was not optimally effective). Local program home care palliative client expenditures compared to the average (regional, provincial, national) in the same time period.   Financial Components: Total publicly funded expenditures including pharmacy costs for the last year of life per home care client. | 29% | 7 |
| **"Cost per patient in hospital generated costs (per home care client per duration of services/per visit). Includes costs associated with readmissions specifically"**   Purpose of the measure: To allow for an understanding of intensity of acute care service provision by home care client. Those clients with more robust and responsive home care services may have reduced lengths of stay resulting in decreased overall expenditures per acute visit.   Financial Components: Funding directly tied to inpatient programming. Includes all inpatient acute care costs (pharmacy, physician, professional, non-professional). | 29% | 3 |
| **"Percent expenditures per local health authority by acute, homecare, public health/mental health)"**  To allow for visibility to the % of spend on home care vs acute services vs community broken down by service areas (public health and mental health). As local health authorities enhance services to community, % spend should shift over time. Additionally, this measure allows for comparability across local health authorities.   Financial Components: Total publicly funded healthcare expenditures proportioned by areas within the continuum of care. Pharmacy, DI, physician billings may be included. | 29% | 5 |
| **"Annual expenditures (home care office)"**   Purpose of the measure: To allow for an understanding of actual annual expenditures for home care programming by office. Example, for visibility in growth year over year.   Financial Components: Funding directly tied to home care programming. Excludes indirect funding (pharmacy for example). | 14% | 3 |
| **"Total (mean) cost per home care client 1 y, 3y and 7y (all publicly funded services) per health authority and/or provincially and/or nationally"**   Purpose of the measure: To understand the actual total expenditures (health authority, municipal, government, etc.) of home care clients on the system (can be compared year over year and across jurisdictions). Can be at regional geography level, provincial and/or nationally. Actual expenditures and growth year over year is an indicator showcasing government/health system commitments or lack of in terms of shifting care to the community. Additionally, this indicator allows for visibility of total cost of care for home care clients within the system.   Financial Components: Total publicly funded expenditures including pharmacy costs. | 14% | 2 |
| **"Total health care costs first 30 days post discharge per home care client"**   Purpose of the measure: To allow for visibility of healthcare usage for the first 30 days post discharge, higher costs may indicate higher levels of care usage (meaning home care was not optimally effective).   Financial Components: Total home care service expenditures per client for the first 30 days post discharge from acute care. | 14% | 5 |
| **"Cost per wound treatment per home care client"**   Purpose of the measure: To allow for an understanding of wound care program operational costs. Wound care can occur in a client's home or in a community ambulatory settings, cost per client allows for visibility of efficiencies of home care programs.   Financial Components: Total cost to treat wounds (home care, medications, supplies, professional). | 14% | 4 |
| **"Home care service cost (mean) per home care client 1 y, 3y and 7y per home care office"**  Purpose of the measure: To understand the service costs by home care office of home care clients for all publicly funded home care specific services over time, how have these changed over the last 1, 3 and 7 years.   Financial Components: Funding directly tied to home care services. Excludes indirect funding (pharmacy for example). | 14% | 2 |
| **"Emergency Department and Acute service cost (mean) per home care client 1 y, 3y and 7y (all publicly funded services) per health authority and/or provincially and/or nationally"**  Purpose of the measure: To understand the actual expenditures (health authority, municipal, government, etc.) of home care clients for acute based services (can be compared year over year and across jurisdictions) over time, how have these changed over the last 1, 3 and 7 years.   Financial Components: Total publicly funded expenditures including pharmacy costs. | 14% | 8 |
| **"Average home care cost per patient per 3 months (total, acute, ED, home care, primary care)"** Purpose of the measure: To understand the actual total expenditures (health authority, municipal, government, etc.) of home care clients on the system (can be compared year over year and across jurisdictions). Can be at regional geography level, provincial and/or nationally. Actual expenditures and growth year over year is an indicator showcasing government/health system commitments or lack of in terms of shifting care to the community. Additionally, this indicator allows for visibility of total cost of care for home care clients within the system.   Financial Components: Total publicly funded expenditures including pharmacy costs. | 14% | 4 |
| **"Difference in total care costs among like clients"**  To compare home care clients to non home care clients or like home care clients from various jurisdictions in their differences in total healthcare expenditures. Based on overall service design and service utilization pathways home care clients utilize higher levels of services (more costly) at a lesser rate than like individuals who are not home care clients, or home care clients who have less home care service programming available.   Financial Components: Total publicly funded healthcare expenditures proportioned by areas within the continuum of care. Pharmacy, DI, physician billings may be included. | 14% | 4 |
| **"Percent change in baseline versus follow up in out of pocket payments for all medical costs in the last month"**  Purpose of the measure: To allow for visibility of patient self-reported financial expenditures on healthcare. Not all health care services are publicly funded, this measure illustrates the amount of out of pocket costs per home care client compared by office, health authority, and province by month.   Financial Components: home care client self reported estimated baseline and follow up in personal money (out of pocket) spent on medical care in the last month. | 14% | 6 |
| **"Home care service cost (mean) per home care client 1y, 3y and 7y (all publicly funded services) per home care office"**   Purpose of the measure: To understand the actual total expenditures by home care office of home care clients for all publicly funded services over time, how have these changed over the last 1, 3 and 7 years.   Financial Components: Funding directly tied to home care clients service utilization. Excludes indirect expenditures (pharmacy for example). | 14% | 2 |
| **"Percent change in baseline vs follow up in money spent in the last month on medical care"**   Purpose of the measure: To allow for an understanding of patient self-reported financial expenditures on healthcare. Not all health care services are publicly funded, this measure illustrates the amount of out of pocket costs per home care client compared by office, health authority, and province.   Financial Components: Home care client self reported estimated baseline and follow up in personal money (out of pocket) spent on medical care. | 14% | 6 |
| **"Percent change in baseline versus follow up in patient caretaker/caregiver lost wages"**  Purpose of the measure: To allow for an understanding of home care patient's caregiver financial burden. A significant component of home care services is the provision of services that act as caregiver supports and caregiver respite. Caregivers provide a significant amount of time and energy to support loved ones, this measure quantifies this support through lost wages.   Financial Components: Home care client self reported estimated baseline and follow up wages. | 14% | 3 |
| **"Population based - Total (hospital, LTC, outpatient and home care) costs per home care patient (6 month)"**   Purpose of the measure: To understanding the actual total expenditures (health authority, municipal, government, etc.) of home care clients on the system (can be compared year over year and across jurisdictions). Can be at regional geography level, provincial and/or nationally. Actual expenditures and growth year over year is an indicator showcasing government/health system commitments or lack of in terms of shifting care to the community. Additionally, this indicator allows for visibility of total cost of care for home care clients within the system.   Financial Components: Total publicly funded healthcare expenditures over a 6 month period per client. | 14% | 4 |
| **"Entire population - mean home care cost per patient (6 month)"**  To allow for visibility of actual total expenditure per home care client (health authority, municipal, government, etc.). Expenditures are mostly tied to services provided, expenditures vary by jurisdiction, health authority and provide. Understanding expenditure variation allows for more understanding of inequities in service delivery.   Financial Components: Funding directly tied to home care programming. Excludes indirect funding (pharmacy for example). | 0% | 5 |
| **"Community based cost (mean) per home care client 1y, 3y and 7y (all publicly funded services) per health authority and/or provincially and/or nationally"**   Purpose of the measure: To understanding the actual total expenditures (health authority, municipal, government, etc.) of home care clients for community based services (can be compared year over year and across jurisdictions) over time, how have these changed over the last 1, 3 and 7 years.   Financial Components: Total publicly funded healthcare expenditures proportioned by areas within the continuum of care. Pharmacy, DI, physician billings may be included. | 0% | 4 |
| **"Assessment of the spending of a home health agency's Post Acute Care Home Health episodes relative to the spending of the national median home health agency's Post Acute Care Home Health episodes across the same performance period"**   Purpose of the measure: To allow for visibility of local program home care client expenditures 30 days total post acute care discharge compared to the average (regional, provincial, national) in the same time period. To allow for visibility of acute and ED usage for the first 30 days post discharge, higher costs indicate higher levels of care usage (meaning home care was not optimally effective).   Financial Components: Total home care service expenditures per client for the first 30 days post discharge from acute care. | 0% | 3 |
| **"Percent change in baseline versus follow up in financial hardship"**   Purpose of the measure: To allow for visibility of the shifts in self reported financial hardship of the patient. Patient reported financial hardship measure as per the interRAI assessment provides information on the ability of home care to provide a full basket of services and supports.   Financial Components: home care client self reported estimated baseline and follow up financial hardship. | 0% | 5 |
| **"Population based - overall community based costs per home care patient overtime (6 month)"**   Purpose of the measure: To allow for comparability of home care expenditures and how these correlate to outpatient expenditures and inpatient expenditures. Home care cost per patient (health authority, municipal, government, etc.).   Financial Components: Funding directly tied to community based programming. Includes all community services (pharmacy, physician, professional, non-professional). Not home care program direct expenses. | 0% | 2 |
| **"Population based - hospital, cost per patient (6 month)"**  Purpose of the measure: To allow for comparability of home care client expenditures and how these correlate to outpatient expenditures and inpatient expenditures. Home care cost per patient (health authority, municipal, government, etc.).   Financial Components: Expenditures directly tied to inpatient programming. Includes all inpatient acute care costs (pharmacy, physician, professional, non-professional). | 0% | 5 |
| **"Population based - outpatient (ambulatory, emergency department) cost per home care patient (6 month)"**   Purpose of the measure: To allow for comparability of home care client expenditures and how these correlate to outpatient expenditures and inpatient expenditures. Home care cost per patient (health authority, municipal, government, etc.). Financial Components: Funding directly tied to outpatient/ambulatory programming such as emergency department visits, diagnostics. | 0% | 5 |
| **"Percent change in baseline vs follow up in lost wages"**   Purpose of the measure: To allow for an understanding of financial hardship for home care patients. A significant component of home care services is the provision of services that act as home supports including caregiver respite. This measure quantifies this support through lost wages of the home care client.   Financial Components: Home care client self reported estimated baseline and follow up wages. | 0% | 3 |
